# *Carex rigescens* caffeic acid O-methyltransferase gene *CrCOMT* confer melatonin-mediated drought tolerance in transgenic tobacco

**DOI:** 10.3389/fpls.2022.971431

**Published:** 2022-08-10

**Authors:** Yan Li, Yan Sun, Huiting Cui, Mingna Li, Guofeng Yang, Zengyu Wang, Kun Zhang

**Affiliations:** ^1^Key Laboratory of National Forestry and Grassland Administration on Grassland Resources and Ecology in the Yellow River Delta, College of Grassland Science, Qingdao Agricultural University, Qingdao, China; ^2^College of Grassland Science and Technology, China Agricultural University, Beijing, China; ^3^Institute of Animal Sciences, Chinese Academy of Agricultural Sciences (CAAS), Beijing, China

**Keywords:** melatonin, COMT, drought stress, transgenic tobacco, *Carex rigescens*

## Abstract

Melatonin is an important, multifunctional protective agent against a variety of abiotic and biotic stressors in plants. Caffeic acid O-methyltransferase (COMT) catalyzes the last step of melatonin synthesis in plants and reportedly participates in the regulation of stress response and tolerance. However, few studies have reported its function in melatonin-mediated drought resistance. In this study, *CrCOMT* was identified and was strongly induced by drought stress in *Carex rigescens*. *CrCOMT* overexpression in transgenic tobacco increased tolerance to drought stress with high levels of seed germination, relative water content, and survival rates. *CrCOMT* overexpression in tobacco improved membrane stability, and plants exhibited lower relative electrolytic leakage and malondialdehyde content, as well as higher photochemical efficiency than the wildtype (WT) under drought stress. The transgenic plants also had higher levels of proline accumulation and antioxidant enzyme activity, which decreased oxidative stress damage due to reactive oxygen species (ROS) hyperaccumulation under drought stress. The transcription of drought stress response and ROS scavenging genes was significantly higher in the *CrCOMT* overexpression plants than in the WT plants. In addition, *CrCOMT* transgenic tobacco plants exhibited higher melatonin content under drought stress conditions. Exogenous melatonin was applied to *C. rigescens* under drought stress to confirm the function of melatonin in mediating drought tolerance; the relative water content and proline content were higher, and the relative electrolytic leakage was lower in melatonin-treated *C. rigescens* than in the untreated plants. In summary, these results show that *CrCOMT* plays a positive role in plant drought stress tolerance by regulating endogenous melatonin content.

## Introduction

Recently, the effects of global climate change, such as increasing temperatures, have increased pressure on plants to cope with increasingly arid environments (Fahad et al., 2017; Dietz et al., 2021). Water has a great impact throughout the plant growth period, and water deficiency can result in plant withering, impaired development, reduced production, and even death (Anjum et al., 2011). Specifically, drought stress can impede root water absorption and leaf stomatal movement, change plant internal water potential, and affect nutrient transport between the aboveground and underground parts of plants (Ahanger et al., 2021). Under drought stress, a high level of reactive oxygen species (ROS) accumulation in plant tissues leads to oxidative peroxidation of cell membranes and oxidative damage to proteins and nucleic acids (Kar, 2011). At the cellular level, there can be damage to the photosynthetic system, decrease in photochemical efficiency, and multiple effects on plant energy metabolism (Dalal, 2021). Therefore, it is crucial to understand the complicated drought stress tolerance mechanisms in plants, which could contribute to improving plant growth in water-deficient soils and developing water-saving agriculture.

Melatonin (N-acetyl-5-methoxytrytamine) was first identified in vascular plants in 1995, and since then, studies have reported multiple functions for plant growth and development (Hattori et al., 1995; Arnao and Hernández-Ruiz, 2019). In addition, over the last two decades, the multifunctional role of melatonin in plants as a protective agent against different abiotic and biotic stressors has been fully accepted, and it is well known to improve tolerance to salt, drought, high temperatures, cold, and heavy metal stress (Hoque et al., 2021; Sun et al., 2021). As a master regulator, melatonin improves plant tolerance to abiotic stress by regulating the expression of downstream genes, osmolyte accumulation, carbon assimilation, stomatal conductance, and photochemical efficiency of photosystems (Sharma and Zheng, 2019; Moustafa-Farag et al., 2020). The most common mechanism of melatonin-mediated anti-stress regulation is the enhancement of plant antioxidant defense activity, such as peroxidase, superoxide dismutase, and catalase, and key soluble osmolytes, such as proline, soluble protein, and sugars (Alharby and Fahad, 2020). In addition, in studies investigating drought stress improvement functions, it was found that exogenous melatonin confers drought stress tolerance by promoting plant growth, photosynthetic capacity, and antioxidant defense system in the tea plant (*Camellia sinensis*) (Li et al., 2019), lemon verbena (*Lippia citriodora*) (Hosseini et al., 2021), tartary buckwheat (*Fagopyrum tataricum*) (Hossain et al., 2020), and soybean (*Glycine max*) (Imran et al., 2021).

N-acetylserotonin methyltransferase (ASMT) participates in melatonin synthesis from N-acetylserotonin in the cytoplasm, however, *ASMT* homology genes in plants are rare (Byeon et al., 2014b; Zhuang et al., 2020). Caffeic acid O-methyltransferase (COMT), an O-methyltransferase, has enzyme activity similar to that of ASMT, and its catalytic function in the last step of melatonin biosynthesis was recently reported (Byeon et al., 2014a; Lee et al., 2017). Recently, the role of *COMT* in melatonin-mediated physiological mechanism variations has been demonstrated in certain plant species. In herbaceous peony (*Paeonia lactiflora*), manipulating the expression of *COMT1*, which is involved in melatonin biosynthesis, had a significant effect on melatonin, S/G ratio, and stem strength (Zhao et al., 2022). In tomatoes, *COMT1* overexpression resulted in increased melatonin biosynthesis and contributed to the alleviation of carbendazim phytotoxicity and residues (Yan et al., 2019). A previous study also showed that OsCOMT encodes caffeic acid O-methyltransferase in melatonin biosynthesis and increases rice (*Oryza sativa*) grain yield through the dual regulation of leaf senescence and vascular development. In addition, the *COMT* gene has been investigated in melatonin-mediated stress resistance (Huangfu et al., 2022). Overexpression of *SlCOMT* in *Solanum lycopersicum* increased melatonin accumulation and improved tolerance of salt stress (Sun et al., 2020). Watermelon *ClCOMT1* has been reported to play an essential role in melatonin biosynthesis and function in abiotic stress tolerance (Chang et al., 2021). A previous study reported that *COMT* gene expression could be affected by water deficit stress, and a recent study in *Sorghum bicolor* had investigated its role in plant drought stress tolerance (Saluja et al., 2021). However, the function of *COMT* in melatonin-mediated plant drought stress tolerance is not well understood.

Turfgrass growth is severely influenced by limited irrigation and drought stress induced by global climate change (Yu et al., 2019; Wang et al., 2021). *Carex rigescens (Franch.) V. Krecz*, a cool season perennial turfgrass, is widely distributed in northern China, has a strong tolerance to various stresses, and can form turf as an ornamental and lawn plant species even in arid and barren soil (Zhang et al., 2020; Zhang K. et al., 2021). Revealing stress resistance mechanisms, such as drought stress tolerance, may be meaningful not only revealing the molecular mechanisms behind *C. rigescens* drought resistance, but also important for drought tolerance genetic breeding in turfgrass and crop plants which could contribute to water-efficient agriculture. The stress induction (including drought stress) performance of *CrCOMT* expression was verified in our previous study, which led us to hypothesize that *CrCOMT* might play a significant role in drought stress tolerance in *C. rigescens* (Zhang et al., 2019). Further investigation into the specific gene function of *CrCOMT* in drought tolerance regulation in *C. rigescens* is required.

In this study, to verify the specific gene function of *CrCOMT* in response to drought stress, the gene expression patterns of *CrCOMT* in different *C. rigescens* tissues and stress conditions were analyzed. *CrCOMT* overexpression transgenic tobacco had been generated, and the role of *CrCOMT* in drought stress tolerance was identified. In addition, we compared seed germination, plant growth, physiological response, and stress-responsive gene expression between transgenic and wildtype (WT) tobacco plants response to drought stress. We also assayed endogenous melatonin content and investigated its role in drought stress tolerance in *C. rigescens*. This study reveals the gene functions and regulatory mechanisms of *CrCOMT* under drought stress conditions.

## Materials and methods

### Plant material and growth conditions

*Carex rigescens* (of the stress tolerant genotype, ‘Huanghua’) seeds were sterilized using 75% ethanol for 30 s and 20% NaOH for 30 min then washed ten times with sterilized water. The seeds were sown on wet filter paper and placed in a growth chamber (BDR16, Conviron) under controlled conditions of 25/20°C and 16/8 h (day/night), 1200-1250 μmol m^–2^s^–1^ light intensity. *C. rigescens* seedlings were then planted in soil (peat soil: roseite = 3:1) for two months in a greenhouse (25/20°C-day and night temperature). Uniform seedlings were then selected and transferred to a container filled with half-strength Hoagland’s nutrient solution, and forty-day-old hydroponic *C. rigescens* seedlings were chosen for further gene expression and stress analysis.

Tobacco (*Nicotiana benthamiana*) seeds were sterilized using 75% ethanol for 30 s and 12% NaClO for 10 min and washed ten times with sterilized water. The seeds were sown on 1/2 Murashig and Skoog (MS) medium and kept in the dark at 4°C for two days before being transferred to a growth chamber for germination. After two weeks, robust young seedlings were planted in soil (peat soil: roseite = 3:1) for further analysis.

### Expression analysis of *CrCOMT* in *Carex rigescens*

To analyze gene expression in different tissues, forty-day-old *C. rigescens* hydroponic seedlings were used, including old leaves (OL), new generation plants (NGP), rhizomes (R), new leaves (NL), root systems (RS), leaf sheaths (LS), and root crowns (RC). To analyze gene expression under stress conditions, forty-day-old *C. rigescens* hydroponic seedlings were separated into the following treatment groups: salt (300 mM NaCl) and drought (30% Polyethylene glycol (PEG)-6000). Leaves were sampled separately at 0, 1, 3, 6, 9, and 12 h after stress treatment. Samples were harvested, immediately frozen in liquid nitrogen, and stored at –80°C for further RNA isolation and gene expression analysis. Each stress treatment was replicated three times.

### Vector construction and tobacco transformation

The *CrCOMT* sequence was acquired based on a previous report (Zhang et al., 2019). First, pCAMBIA3301 was constructed as the expression vector (*Nco*I*/Bgl*II) using In-Fusion^®^ Snap Assembly Master Mix (Takara, China) (Primer: CrCOMT-p3301-S/CrCOMT-p3301-A). The recombinant vector pCAMBIA3301-*CrCOMT* was introduced into *Agrobacterium tumefaciens* strain EHA105. Tobacco agrobacterium-mediated transformation was performed with tobacco leaf disk infiltration, according to the protocol in Gallois and Marinho’s study (Gallois and Marinho, 1995). T_0_ transgenic tobacco seeds were selected on 1/2 MS medium containing phosphinothricin (50 mg/L). Positive transgenic seedlings were identified using PCR (Primer: 35S-specific-S/CrCOMT-specific-A). Then, qRT-PCR was performed, transgenic tobacco plants with high expression were selected (Primer: qCrCOMT-S/qCrCOMT-A), and homozygous T_3_ seeds were used for further experiments. The primers used in PCR and qRT-PCR were designed using Primer 5 software and detailed sequence were shown in Supplementary Table 1.

### Transgenic tobacco stress tolerance validation

Transgenic and WT tobacco seeds were sterilized and germinated on 1/2 MS with NaCl (100 and 150 mM), mannitol (250 and 275 mM), or no stressor. Germination was recorded based on the emergence of the radicle tip in tobacco seed. Each treatment had three independent replicates and at least 100 tobacco seeds per replicate. For the drought tolerance test, (1) transgenic and WT tobacco plants were grown in soil (peat soil: roseite = 3:1) for four weeks, then tobacco seedlings were treated with 15% PEG-6000 for 3 days. Leaves samples were collected for gene expression analysis. When tobacco plants exhibited lethal effects of dehydration, watering was resumed and the tobacco plants were recovered for 3 days. The photographs were taken after PEG-6000 treatment and rewatering. (2) transgenic and WT tobacco plants were grown in soil (peat soil: roseite = 3:1) for four weeks with sufficient watering. The plants were then subjected to drought stress by withholding irrigation. All pots were placed under the same conditions in a growth chamber, and the position of each pot was randomly changed every day to exclude any positional effects. Photographs were taken at 6 and 12 days after withholding the water treatment, and the survival rate was calculated. Twelve days after the beginning of stress treatment, the leaves were harvested for physiological index analysis. For salt-sensitivity assays in soil, transgenic and WT tobacco plants were grown in soil (peat soil: roseite = 3:1) under long-day conditions for four weeks. Subsequently, the soil was irrigated with NaCl solution every three days. The initial concentration was 50 mM, which was increased by 50 mM per day to a final concentration of 200 mM. After 21 days of 200 mM NaCl treatment, the tobacco plants were photographed.

### Quantitative RT-PCR analysis

Total RNA was isolated using a MiniBEST Plant RNA Extraction Kit (Takara, China), following the manufacturer’s instructions. Total RNA (1 μg) was used for cDNA synthesis using the PrimeScriptTM RT Reagent Kit with gDNA Eraser (Takara, China). qRT-PCR was performed on a CFX96 Touch Real-Time PCR Detection System (BIO-RAD, United States) with TB Green^®^ Premix Ex Taq™ (Tli RNaseH Plus; Takara, China) according to the manufacturer’s instructions. The primers used in qRT-PCR were designed using Primer 5 software and detailed sequence were shown in Supplementary Table 1. The *CreIF-4*α gene was used as a reference control for *CrCOMT* expression patterns in *C. rigescens* according to our previous study (Zhang et al., 2020), and *NtActin* was used as a reference in all tobacco qRT-PCR analyses. Relative gene expression was calculated using the 2^–ΔΔ*Ct*^ method (Livak and Schmittgen, 2001) and the results are presented in terms of fold-change.

### Melatonin assay

Melatonin content in tobacco plants under normal and drought stress conditions was determined according to a previously described method with an ELISA kit, as described by Zhang (Zhang et al., 2017). To determine the potential effect of exogenous melatonin application on *C. rigescens*, forty-day-old hydroponic *C. rigescens* seedlings were pre-sprayed with 50 μM melatonin before exposure to drought stress. After seven days of melatonin treatment, drought stress treatment was applied by adding 15% PEG-6000 to half-strength Hoagland nutrient solution. Leaf samples were collected after seven days of drought treatment for further relative water content, relative electrolytic leakage, and proline content measurement. Each treatment was replicated three times.

### Physiological measurements

To measure leaf photochemical efficiency, leaves were dark-adapted for 30 min before measurement, and variable fluorescence (Fv)/maximal fluorescence (Fm) was measured using a FluorPen FP110 chlorophyll fluorescence meter (PSI, Czechia). To measure relative water content, 0.2 g leaf tissue (W0) was sampled and soaked in a tube containing 30 ml distilled water for 4 h. Leaf surface water was dried with a paper towel and weighed (W1). Following this, the leaf tissues were dried at 80°C and weighed (W2). The relative water content was calculated as (W1-W0/W2-W0) × 100. To examine relative electrical conductivity, 0.2 g leaf tissue (W0) was sampled and soaked in a tube containing 30 ml distilled water for 4 h at 25°C, then conductance (R0) was measured with a conductivity meter (FE38, Mettler Toledo). Afterward, the tubes were autoclaved at 121°C for 20 min. After cooling to 25°C, the final conductance (R1) was measured. Relative electrical conductivity was calculated as R0/R1 × 100. To measure chlorophyll content, 0.1 g leaf tissue was sampled and soaked in a tube containing 10 ml dimethyl sulfoxide for two days, and the extracted chlorophyll was measured at 663 nm and 645 nm using a spectrophotometer (UV-2700, Shimadzu). The chlorophyll content was calculated as described previously (Barnes et al., 1992).

Proline content was measured using an acidic ninhydrin-based method (Ábrahám et al., 2010) with a colorimetric PRO assay kit (BC0290, Solarbio Life Sciences, China), and the absorbance was detected at a wavelength of 520 nm using a spectrophotometer. Malondialdehyde (MDA) content was determined using the thiobarbituric acid (TBA)-based colorimetric method (Wang et al., 2013) with a colorimetric MDA assay kit (BC0020, Solarbio Life Sciences, China), and absorbance was detected at a wavelength of 532 nm. Catalase (CAT) activity was measured using an H_2_O_2_ -based method (Scebba et al., 2001) colorimetric CAT Kit (BC0200, Solarbio Life Sciences, China), and absorbance was detected at a wavelength of 240 nm. Peroxisome (POD) activity was measured using the guaiacol method (Zhang and Kirkham, 1996) with a colorimetric POD Kit (BC0090, Solarbio Life Sciences, China), and absorbance was detected at a wavelength of 470 nm. Superoxide dismutase (SOD) activity was measured using a nitroblue tetrazolium (Giannopolitis and Ries, 1977) colorimetric SOD Kit (BC0170, Solarbio Life Sciences, China), and absorbance was measured at 560 nm.

### Statistical analysis

The data in this study were subjected to statistical analyses using SPSS v20.0 (SPSS Inc., United States). Significant differences were determined using Fisher’s protected least significant difference test at a probability level of 0.05. *P* < 0.05 was consider statistically significant.

## Results

### Expression profile analysis of *CrCOMT* in *Carex rigescens*

**FIGURE 1:**
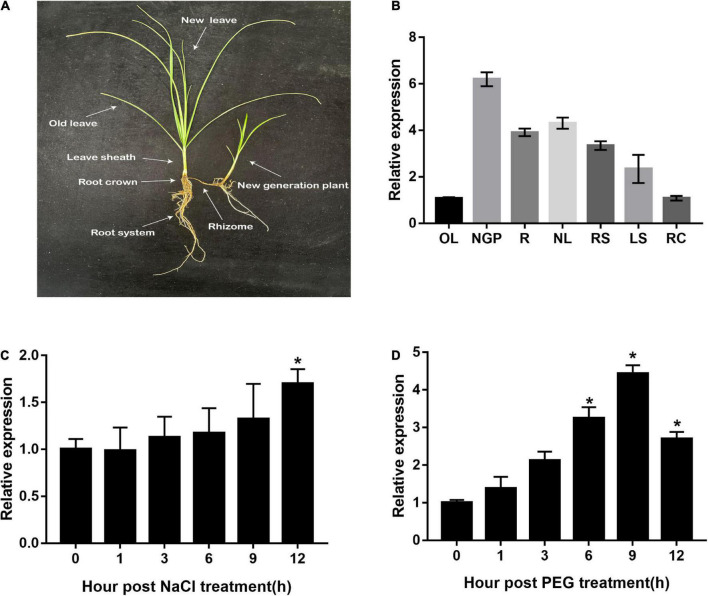
*CrCOMT* expression profile in different tissues and stress conditions in *C. rigescens*. **(A)** *C. rigescens* different tissues including old leave (OL), new generation plant (NGP), rhizome(R), new leave (NL), root system (RS), leave sheath (LS), and root crown (RC). **(B)** *CrCOMT* gene expression in *C. rigescens* different tissues. **(C,D)** Drought stress was used of 30% PEG-6000 and salt stress was used of 300 mM NaCl. *C. rigescens* leaves were harvested separately at different time points after stress treatment for qRT-PCR analysis. *CreIF-4*α gene was used as reference gene. All data are means ± SE for three biological replicates. * Over the bars indicated the significance at *P* < 0.05 among different times points.

Different tissues, as well as salt- and drought-treated leaves of *C. rigescens* seedlings, were harvested to explore the expression pattern of *CrCOMT*. Specifically, *C. rigescens* different tissues were separately displayed in Figure 1A. *CrCOMT* was expressed at a higher level in new-generation plants (6.20 fold), rhizomes (3.90 fold), new leaves (4.31 fold), root systems (3.34 fold), and leaf sheaths (2.33 fold), and lower levels in old leaves (1.06 fold) and root crowns (1.08 fold; Figure 1B). For the stress treatment, *CrCOMT* was slightly induced within 12 h of NaCl treatment and significantly increased and reached a maximum level at 12 h after treatment (1.70 fold) (Figure 1C); under PEG treatment, *CrCOMT* expression was significantly increased at 6 (3.25 fold), 9 (4.43 fold), and 12 (2.70 fold) h after treatment, with the highest expression level at 9 h (Figure 1D).

### Germination rate of *CrCOMT* transgenic tobacco seeds under different stress conditions

**FIGURE 2:**
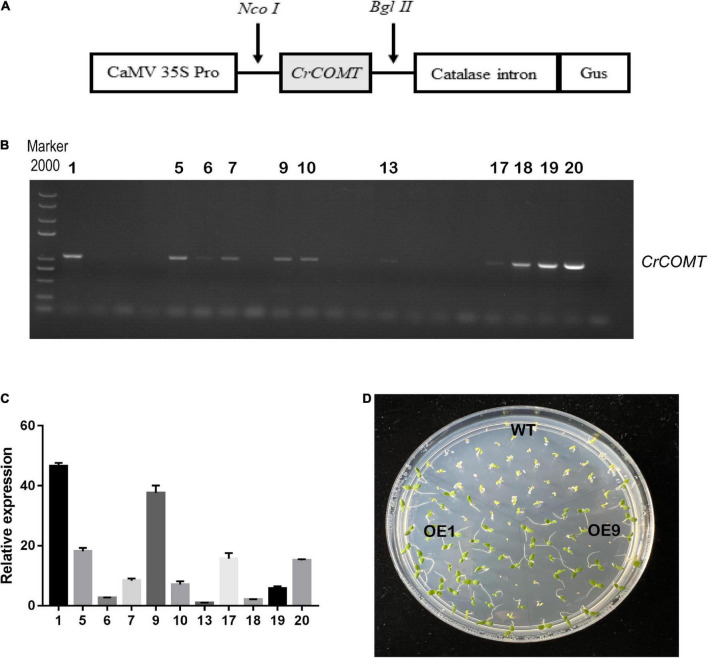
*CrCOMT* overexpression transgenic plants screening and evaluation. **(A)** Expression vector construction of *CrCOMT*. **(B)** Positive transgenic identified by agarose gel electrophoresis. **(C)** Transcription levels of *CrCOMT* in different positive overexpression tobacco plants. **(D)** Transgenic tobacco OE1 and OE9 had highest expression level were choose by antibiotic and homozygous lines were used for further analysis. The *NtActin* gene was used as internal controls. All data are means ± SE for three biological replicates.

**FIGURE 3:**
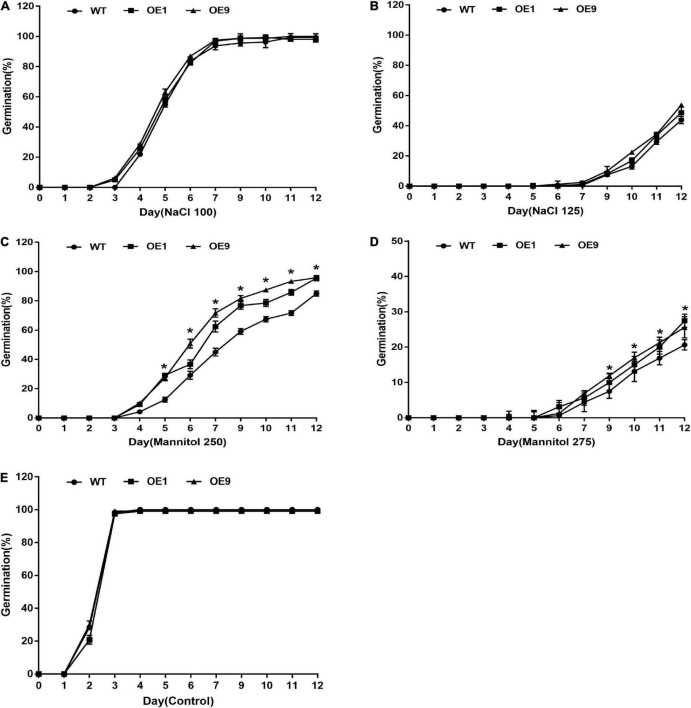
Seed germination of transgenic tobacco plants under stress conditions for 12 days. Seed germination rate of OE1, OE9, and WT tobacco plants under **(A)** 100 mM NaCl, **(B)** 125 mM NaCl, **(C)** 250 mM Mannitol, **(D)** 275 mM Mannitol, and **(E)** Control treatment. All data are means ± SE for three biological replicates.

To characterize gene function, a *CrCOMT* overexpression vector was constructed (Figure 2A) and different *CrCOMT* overexpression transgenic tobacco plants were generated and detected using PCR and quantitative PCR (Figures 2B–C). Two transgenic tobacco lines (OE1 and OE9) with high expression levels were selected (Figure 2D). Then, OE1, OE9, and WT tobacco T_3_ seeds were cultured on 1/2 MS medium with NaCl (100 and 150 mM), mannitol (250 and 275 mM), or no stressor; the germination rates were counted and are shown in Figure 3. Specifically, under the 100 mM NaCl treatment, there was no significant difference between the transgenic and WT tobacco plant seed germination rates. When treated with 125 mM NaCl, the OE9 germination rate was higher than that of OE1 and WT after ten days of germination (Figures 3A,B). For the drought stress treatment, the seed germination rate of the two transgenic lines was significantly higher than that of the WT after four days of germination under 250 mM mannitol treatment. Additionally, the seed germination rates of the two transgenic tobacco plants were significantly higher than those of the WT plants after seven days of 275 mM mannitol treatment (Figures 3C,D). The seed germination rate of transgenic and WT plants both reached 100% after two days without a stressor (Figure 3E). These results suggest that *CrCOMT* may significantly improve transgenic plant drought resistance; therefore, the gene function in drought tolerance was further studied.

### *CrCOMT* enhanced the drought tolerance of transgenic tobacco plants

**FIGURE 4:**
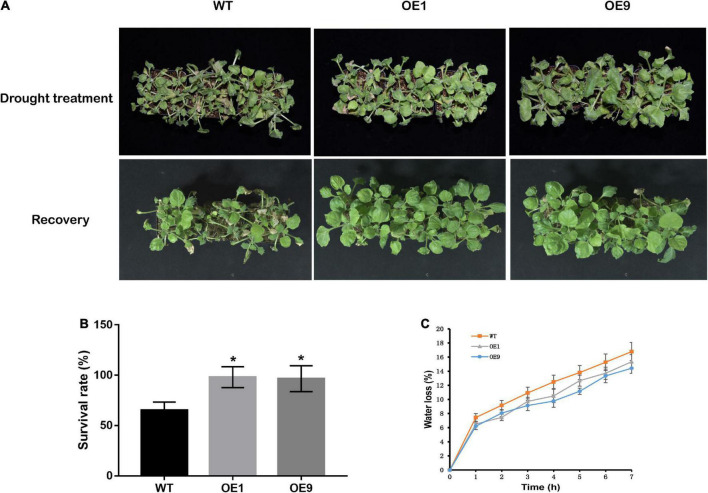
Physiological performance of transgenic tobacco plants under drought stress condition. **(A)** The phenotype of tobacco plants after PEG-6000 treatment and rewatering. **(B)** Survival rate of tobacco plants after rewatering. **(C)** For leaves water loss measurement, transgenic and WT tobacco plants were grown in soil (peat soil: roseite = 3:1) for four weeks, then leaves were detached and placed on a filter paper in growth chamber. Leaves were weighed at designated time points, and the loss of fresh weight (percentage) was used to indicate water loss. All data are means ± SE for three biological replicates. * Over the bars indicated the significance at *P* < 0.05 between WT and transgenic tobacco plants.

To verify whether *CrCOMT* overexpression alters plant drought stress resistance, drought sensitivity and tolerance assays of transgenic and WT tobacco plants were performed. For the drought sensitivity assay, four-week-old transgenic and WT plants were treated with 15% PEG-6000 for seven days and rewatering for three days (Figure 4A). After treatment, both transgenic and WT tobacco displayed a leaf dehydration phenotype after PEG-6000 treatment, but WT tobacco leaves showed more significant withering than transgenic plants. After rewatering, only part of each WT tobacco plant recovered, compared to the majority of transgenic tobacco plants. Following this, the survival rate of drought stress treated tobacco plants was counted, which was higher in both transgenic plants OE1 (98.06%) and OE9 (96.59%) than in the WT plants (65.27%; Figure 4B). For the detached leaf dehydration trial, the water loss of OE1 and OE9 was lower than that of the WT within 7 h after dehydration treatment (Figure 4C), indicating that *CrCOMT* overexpression plants retained more water in detached leaves.

**FIGURE 5:**
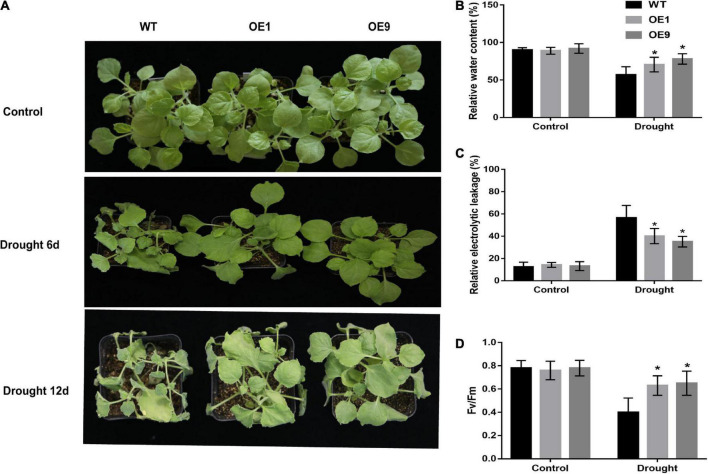
Expression of *CrCOMT* enhanced drought tolerance in transgenic tobacco plants. **(A)** The phenotype of four-week-old transgenic and WT tobacco plants under control and drought stress after withholding irrigation. Each experiment had four plant tobacco plants in one plot. The experiment had four replicates. **(B-D)** Relative water content, relative electrical conductivity and Fv/Fm value in transgenic and WT tobacco plants under control and drought stress conditions were measured. All data are means ± SE for three biological replicates. * Over the bars indicated the significance at *P* < 0.05 between WT and transgenic tobacco plants.

For the natural drought stress experiment, four-week-old transgenic and WT plants were subjected to irrigation. After six days of withholding irrigation, the WT plants started to wither while the transgenic plants remained normal, whereas after 12 days of withholding irrigation, WT tobacco plants displayed a more significantly withered phenotype than transgenic tobacco plants (Figure 5A). In transgenic tobacco plants, the relative water content and Fv/Fm ratio were significantly higher, and leaf electrolyte leakage was significantly lower than that in WT plants (Figures 5B–D). A non-significant difference in these indices was observed between the transgenic and WT tobacco plants under normal conditions. We also subjected four-week-old transgenic and WT tobacco plants to 200 mM NaCl treatment (Supplementary Figure 1). After 21 days of treatment, there was no significant difference between *CrCOMT* overexpression plants and WT plants, but more old-yellow leaves were observed in the WT plants.

**FIGURE 6:**
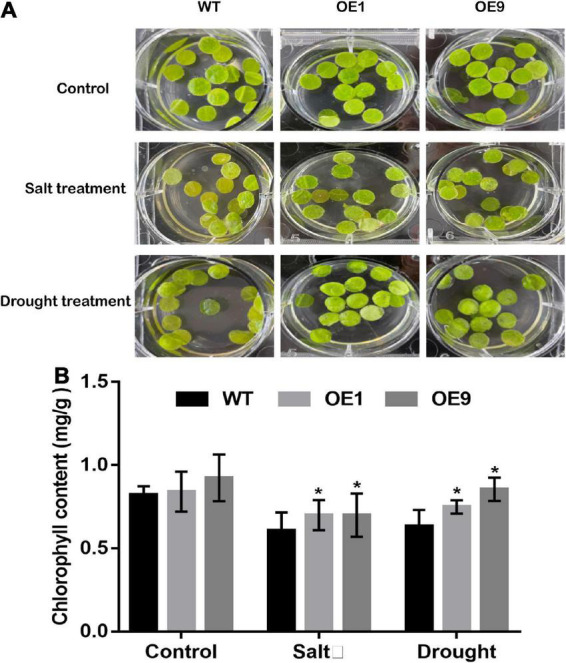
Leaf dish phenotype assay of transgenic tobacco plants under stress conditions. **(A)** Leaf disks from five-week-old transgenic and WT tobacco plants were detached and floated on 1/2 MS, 1/2 MS with 300 mM NaCl, and 300 mM Mannitol for 7 days, then the leaf disks was photographed **(B)** Chlorophyll content of OE1, OE9, and WT tobacco plant leaf dish measured. All data are means ± SE for three biological replicates. * Over the bars indicated the significance at *P* < 0.05 between WT and transgenic tobacco plants.

Furthermore, a leaf disk assay was performed using 300 mM NaCl and 300 mM mannitol to assess the stress tolerance of mature transgenic tobacco leaves. The phenotypes of transgenic and WT tobacco leaf disks after stress treatment are shown in Figure 6A. Meanwhile, the chlorophyll content was measured, and transgenic tobacco plants displayed lower levels of degradation than WT plants (Figure 6B). In summary, transgenic tobacco plants showed higher drought tolerance than WT plants based on different drought stress assays.

### Stress marker substance and antioxidant enzyme variation in *CrCOMT* transgenic tobacco plants

**FIGURE 7:**
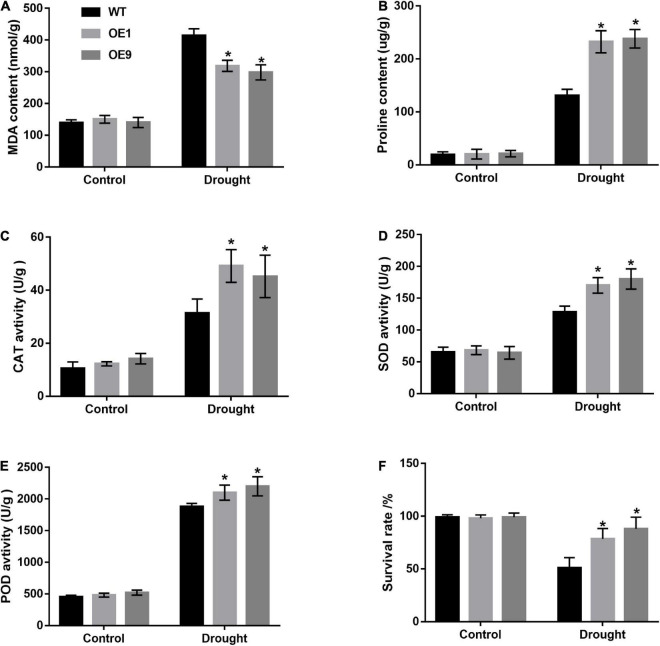
Analysis of increased drought tolerance in *CrCOMT* overexpression transgenic tobacco plants. **(A)** MDA content, **(B)** Proline content, the activity of **(C)** CAT enzyme, **(D)** SOD enzyme, **(E)** POD enzyme, and **(F)** survival rate in transgenic and WT tobacco plants as described in Figure 5A were measured. All data are means ± SE for three biological replicates. * Over the bars indicated the significance at *P* < 0.05 between WT and transgenic tobacco plants.

Stress-related substances, such as MDA and proline, are recognized markers for plant drought stress tolerance evaluation. Under normal conditions, the MDA and proline content of *CrCOMT* overexpression tobacco plants was not significantly different from the WT plants (Figures 7A,B). After drought stress treatment, the *CrCOMT* overexpression plants showed significantly lower MDA and higher proline content than the WT tobacco plant (Figures 7A,B), which indicated better drought resistance performance. Antioxidant enzyme activation is important for improving plant ROS-scavenging and drought stress resistance. CAT, SOD, and POD enzyme activity levels were significantly higher in transgenic tobacco plants than in WT plants, but the difference was not significant in the treatment without stressors (Figures 7C–E). In addition, the survival rate of tobacco plants without irrigation was determined (Figure 7F); this was higher in transgenic tobacco plants than in WT plants under the influence of stressors but was not significantly different without stressors. Overexpression of *CrCOMT* could alleviate the damage caused by drought stress via enhancing the ability of osmotic regulation and activity of antioxidant enzymes in tobacco plants.

### Analysis of drought stress related genes in *CrCOMT* transgenic tobacco plants

**FIGURE 8:**
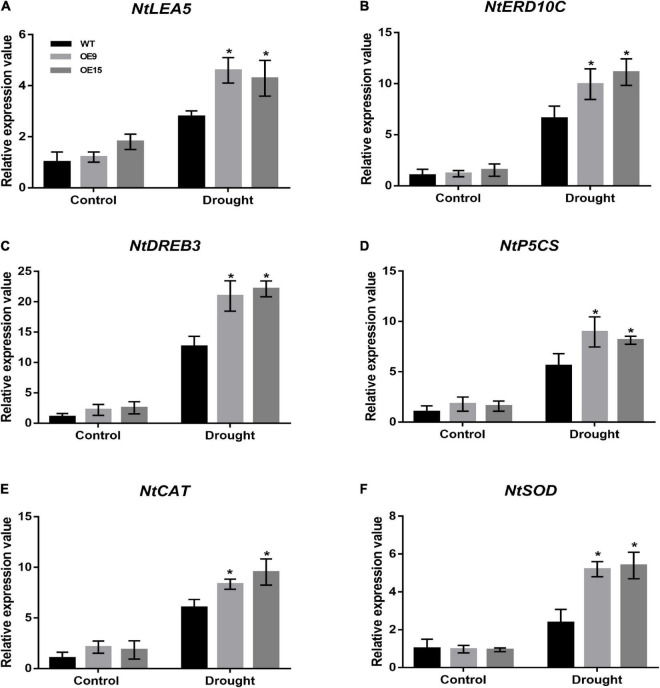
Expression of drought stress related genes in transgenic tobacco plants. Transcription levels of stress response and ROS scavenging genes (*NtLEA5*, *NtERD10C*, *NtDREB3*, *NtP5CS*, *NtCAT*, and *NtSOD*) in transgenic and WT tobacco plants as described in as described in Figure 4A were determined. Untreated plants were used as controls. Expression of the *NtActin* gene was used as an internal control. All data are means ± SE for three biological replicates. * Over the bars indicated the significance at *P* < 0.05 between WT and transgenic tobacco plants.

In response to environmental stress, plants modulate the expression of many stress-response genes, constituting an important molecular basis for stress responses and adaptations in plants. To elucidate the molecular regulatory mechanisms of *CrCOMT*-mediated drought stress tolerance, we analyzed the variations in expression of four drought stress response genes (*NtLEA5*, *NtERD10C*, *NtDREB3*, and *NtP5CS*; Figures 8A–D) and two ROS scavenging genes (*NtCAT* and *NtSOD*; Figures 8E,F). Specifically, the drought stress response and ROS scavenging genes were not significantly different between transgenic and WT tobacco in the absence of stressors (Figures 8A–F). Nevertheless, these genes were all highly induced in both transgenic and WT plants and showed significantly higher expression in the *CrCOMT* overexpression plants than in the WT plants after drought stress treatment (Figures 8A–F). These results suggest that *CrCOMT* can increase drought stress tolerance in transgenic tobacco plants by regulating the stress response and ROS scavenging gene expression.

### Melatonin content in *CrCOMT* transgenic tobacco plants and validation of its role in improving drought tolerance in *Carex rigescens*

**FIGURE 9:**
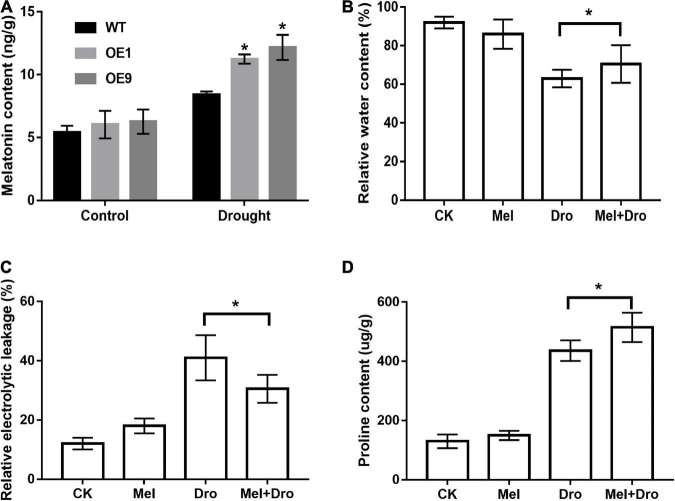
*CrCOMT* affect melatonin synthesis in transgenic tobacco plants and melatonin confers drought tolerance in *C. rigescens*. **(A)** The melatonin content of transgenic and WT tobacco under control and drought treatment. All data are means ± SE for three biological replicates. * Over the bars indicated the significance at *P* < 0.05 between WT and transgenic tobacco plants. **(B)** Relative water content, **(C)** relative electric conductivity and **(D)** proline content was measured after seven days of drought stress in *C. rigescens*. CK, control optimal condition; Mel, melatonin treatment; Dro, drought stress; Mel + Dro, melatonin treatment + drought stress. All data are means ± SE for three biological replicates. * Over the bars indicated the significance at *P* < 0.05 between different melatonin treatment.

It is well documented that the *COMT* gene plays an important role in the plant melatonin synthesis pathway and might participate in plant stress tolerance. To verify the function of *CrCOMT* in drought stress tolerance improvement, endogenous melatonin content was measured in transgenic and WT tobacco plants. Under normal conditions, endogenous melatonin levels were not significantly different between the transgenic and WT tobacco plants (Figure 9A). However, there was a significant increase in transgenic tobacco plants compared to WT plants under drought stress conditions and the endogenous melatonin content in OE1, OE9 transgenic, and WT tobacco plants were 11.23, 12.16, and 8.4 ng/g, respectively (Figure 9A), which indicates that *CrCOMT* overexpression could increase melatonin biosynthesis in transgenic tobacco plants under drought stress conditions.

To evaluate the drought tolerance function of melatonin in *C. rigescens*, *C. rigescens* plants were pretreated with exogenous melatonin and then subject to PEG-6000 treatment. After seven days drought treatment, melatonin treatment slightly decreased relative water content and increased relative electrolytic leakage under non-stress conditions, but the difference was not significant (Figures 9B,C). Under drought stress conditions, melatonin treatment resulted a significant increase in the relative water content and proline content compared to the untreated plants (Figures 9B,D). In addition, compared to the non-melatonin treatment, the relative electrolytic leakage in the melatonin treatment group was significantly decreased after drought stress (Figure 9C). In the absence of stressors, there was no significant difference between the control and melatonin treatments in proline content (Figure 9D).

## Discussion

Plant O-methyltransferase can transform the methyl group in S-adenosyl-L-methionine to different metabolites, such as flavonoids, alkaloids, and phenols, which play important roles in plant growth, signal transduction, and abiotic and biotic responses (Lam et al., 2007; Struck et al., 2012). Plant O-methyltransferase can be divided into two groups according to their protein molecular weight: class I (23-29 KDa) proteins, which mainly function in lignin synthesis and metabolism, and class II (38-43 KDa) proteins, which mainly affect the metabolism of metabolites, such as flavone, melatonin, and lignin, and participate in plant stress response and tolerance (Gang et al., 2002). CrCOMT is a plant O-methyltransferase class II protein, with a predicted molecular weight of approximately 40.196 KDa (Zhang et al., 2019). Recently, the role of COMT in melatonin biosynthesis has been demonstrated in several species (Byeon et al., 2014a,b), however, few studies have reported the participation of class II O-methyltransferases in plant drought resistance. Based on gene expression under stress conditions, we hypothesized that *CrCOMT* participates in the response of *C. rigescens* to salt and drought stress. To test this, *CrCOMT* was overexpressed in transgenic tobacco and its gene function in response to salt and drought stress was characterized. The transgenic tobacco showed potentially improved drought stress tolerance compared to salt stress, according to a germination trial. Further studies on growth performance and physiological and biochemical reactions to drought stress in transgenic plants suggest *CrCOMT* might play a positive role in dehydration stress tolerance, and this provides a fundamental basis for research on gene regulatory mechanisms.

Drought stress-induced ROS hyperaccumulation results in severe oxidative damage to plant membrane lipids and plant-related electrolytic leakage can be used as a membrane injury indicator to partially reflect cellular membrane lipid peroxidation (Ferreira et al., 2021; Kumar et al., 2021). In this study, transgenic tobacco had a lower lipid peroxidation level than WT plants after drought stress treatment, based on relative electrolytic leakage measurements. The accumulation of MDA reflects the degree of damage suffered by plant cells and the degree of membrane lipid damage (Kumar et al., 2021). After drought stress treatment, the MDA content was lower in the transgenic tobacco plants than in the WT plants, illustrating that *CrCOMT* overexpression could alleviate the degree of membrane lipid damage in tobacco. Plant photosynthetic membrane systems also undergo destruction after drought stress, leading to decreased photochemical efficiency. In the present study, transgenic tobacco plants displayed higher photochemical efficiency and lower degradation than WT plants, according to chlorophyll content measurement and leaf disk observation after drought stress treatment. In summary, *CrCOMT* overexpression may help maintain plant membrane lipid stability under drought stress conditions.

To alleviate oxidative stress damage under drought stress conditions, plant cells simultaneously initiate a series of response mechanisms and stress signals, such as the activation of cellular ROS scavenging mechanisms, which can trigger the production of ROS scavenging enzymes and antioxidants, including SOD, POD, and CAT (Reddy et al., 2004). In this study, there was a marked increase in POD, SOD, and CAT activity levels in transgenic lines compared to WT plants under drought stress treatment, and the corresponding gene expression levels variation of *NtSOD* and *NtCAT* were also consistent with the SOD and CAT activities. Similar ROS scavenging enzyme responses have been reported in *SlCOMT1* overexpression plants, as they significantly enhance antioxidant capabilities, with higher antioxidant enzyme activity observed, including superoxide dismutase, peroxide, and catalase activity, under salt stress conditions (Sun et al., 2020). In addition, overexpression of *COMT1* enhanced antioxidant enzymatic activity and the capacity of tomato plants to reduce MBC phytotoxicity and residues (Yan et al., 2019). Taken together, these results indicate that *CrCOMT* may function to mediate the transcriptional upregulation of ROS-scavenging genes to enhance ROS-scavenging ability and improve drought stress tolerance.

Previous studies have shown the involvement of *COMT* in stress tolerance associated with the regulation of stress response gene expression (Vincent et al., 2005; Zhang X. et al., 2021). Molecular chaperones play crucial roles in osmotic adjustment in plants under drought stress, and LEA5 and ERD10C encode late embryogenesis abundant proteins, which partially bind water, help stabilize enzymes and macromolecular structures, and reduce membrane damage (Hundertmark and Hincha, 2008; Magwanga et al., 2018). The dehydration-responsive element-binding protein (DREBs) transcription factor comprises many stress-responsive regulatory genes, and *DREB3* is important for regulating drought stress responses and is used as a marker gene in many plant drought stress tolerance studies (Umezawa et al., 2006). In this study, the transcript abundance of drought-induced protein genes, such as *NtLEA5* and *NtERD10C* and drought stress-responsive gene, such as *NtDREB3* were higher in transgenic tobacco than in the WT after drought stress, suggesting that these genes were upregulated by *CrCOMT* under drought conditions. In addition, 1-pyrroline 5-carboxylate synthetase (P5CS), the rate-limiting enzyme in proline biosynthesis in plants, can control proline levels, which are critical in improving the stress tolerance of plants (Rai and Penna, 2013). The *NtP5CS* gene was found to be more highly induced in *CrCOMT* transgenic tobacco than in WT plants, and its expression was also consistent with the proline content variation. These results highlight the interconnection between *CrCOMT* overexpression and drought stress tolerance, as it results in the increased expression of several stress-responsive genes.

Melatonin is a multifunctional regulator in plant response to drought stress. (1) The accumulation of osmolytes, such as free proline, were enhanced in melatonin-treated plants, significantly alleviating adverse effects by facilitating the maintenance of optimum turgor pressure (Zhao et al., 2021). (2) Melatonin induces the expression of transcription factors and modulates the expression of downstream stress-responsive genes, thereby increasing plant drought tolerance (Sharma and Zheng, 2019). (3) Melatonin also could regulate the key genes encoding antioxidative enzymes and activate the activities of these enzymes, which maintain redox balance in cells under stressful conditions. The regulation of transcript levels of key genes encoding antioxidative enzymes has been reported in melatonin-mediated drought stress regulation in chinese hickory (*Carya cathayensis*) (Sharma et al., 2020), maize (*Zea mays*) (Ahmad et al., 2019), and kiwifruit (*Actinidia chinensis*) (Xia et al., 2020). According to the results of the current study, melatonin content variation can partially explain the morphological, physicochemical, and molecular functions in *CrCOMT* overexpression transgenic tobacco under drought stress. To further verify the function of melatonin in mediating drought tolerance, we applied exogenous melatonin to *C. rigescens* under drought stress. The relative water content and proline content were higher, and the relative electrolytic leakage was lower in melatonin-treated *C. rigescens* than in the untreated plants under drought conditions. Therefore, we determined that melatonin may play a positive role in the drought stress tolerance of *C. rigescens*.

In summary, we report a class II O-methyltransferase gene, *COMT*, in *C. rigescens* that plays a positive role in plant drought stress tolerance, which may be associated with its gene function in melatonin synthesis. Genetic functional analysis of *CrCOMT* provides a potential candidate gene for drought tolerance mechanisms. Further analyses of the up- and downstream regulatory genes of *CrCOMT* and its enzyme characteristics in melatonin metabolism will help to enrich the understanding of plant *COMT* gene regulatory mechanisms under drought stress.

## Data availability statement

The raw data supporting the conclusions of this article will be made available by the authors, without undue reservation.

## Author contributions

KZ and YS carried out the experimental design. YL, KZ, ML, and HC performed the experiments. YL, KZ, and YS prepared the manuscript and coordinated its revision. KZ, GY, and ZW read and revised the manuscript. All authors provided helpful discussions and approved its final version.

## Supplementary material

Supplementary Figure 1 Phenotype of transgenic and WT tobacco plants under salt stress treatment. Four-week-old transgenic and WT tobacco seedlings were irrigated with 200 mM NaCl solution. The photographs were taken after three weeks salt stress treatment. Six plants were grown in one plot and each experiment had four replicates.
