# Supplementary figures and images for: Carex rigescens caffeic acid O-methyltransferase gene CrCOMT confer melatonin-mediated drought tolerance in transgenic tobacco
Source: Front Plant Sci. 2022 Aug 10;13:971431. doi: 10.3389/fpls.2022.971431 (PMC9399801; doi:10.3389/fpls.2022.971431)

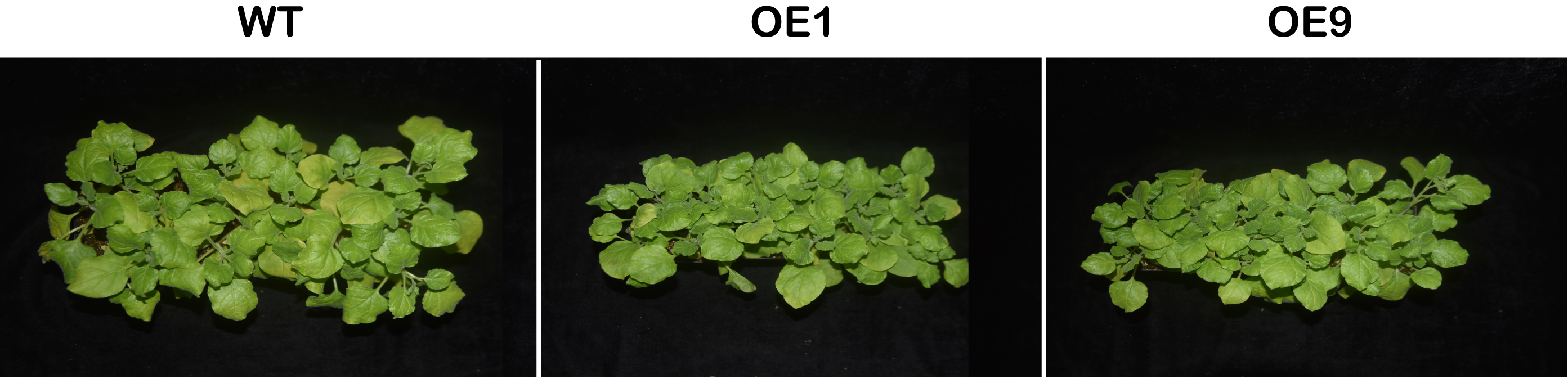

Supplement: Supplementary Figure 1 — Phenotype of transgenic and WT tobacco plants under salt stress treatment. Four-week-old transgenic and WT tobacco seedlings were irrigated with 200 mM NaCl solution. The photographs were taken after three weeks salt stress treatment. Six plants were grown in one plot and each experiment had four replicates. [file Image_1.TIF]
